# Synergizing Nanosensor-Enhanced Wearable Devices with Machine Learning for Precision Health Management Benefiting Older Adult Populations

**DOI:** 10.1021/acsnano.5c04337

**Published:** 2025-07-14

**Authors:** Zhihao Li, Bangshun He, Yiwei Li, Bi-Feng Liu, Guojun Zhang, Songlin Liu, Tony Ye Hu, Ying Li

**Affiliations:** † School of Laboratory Medicine, Engineering Research Center of TCM Protection Technology and New Product Development for the Elderly Brain Health, Ministry of Education, 71045 Hubei University of Chinese Medicine, 16 Huangjia Lake West Road, Wuhan 430065, China; ‡ 240515 Hubei Shizhen Laboratory, 16 Huangjia Lake West Road, Wuhan 430065, China; § Department of Laboratory Medicine, Nanjing First Hospital, 12461 Nanjing Medical University, Nanjing 210006, China; ∥ The Key Laboratory for Biomedical Photonics of MOE at Wuhan National Laboratory for Optoelectronics—Hubei Bioinformatics & Molecular Imaging Key Laboratory, Systems Biology Theme, Department of Biomedical Engineering, College of Life Science and Technology, 12443 Huazhong University of Science and Technology, Wuhan 430074, China; ⊥ College of Traditional Chinese Medicine, Hubei University of Chinese Medicine, 16 Huangjia Lake West Road, Wuhan 430065, China; # Center for Cellular and Molecular Diagnostics, Tulane University School of Medicine, New Orleans, Louisiana 70112, United States; ∇ Department of Biochemistry and Molecular Biology, Tulane University School of Medicine, New Orleans, Louisiana 70112, United States

**Keywords:** wearable device, physiological, biochemical, nanosensor, machine learning, big data, health management, older adults

## Abstract

Population aging presents significant health challenges and socioeconomic burdens globally, driving an increased demand for precision health management. In the era of big data, the exponential growth of health information is accelerating advances in precision health strategies for older adults. For this population, effective strategies can be achieved by the integration of wearable devices, nanosensors, and machine learning. Wearable devices enable continuous monitoring of diverse, real-time health metrics, serving as vital tools for collecting comprehensive health data. Nanosensors can be loaded into wearable devices to enhance their performance by significantly improving detection sensitivity and specificity, thereby increasing the accuracy and reliability of the data collected. Meanwhile, machine learning provides powerful methods for rapid and efficient analysis of large-scale health data, driving the optimization of nanosensors as well as wearable devices. This review examines the synergistic roles of wearable devices, nanosensors, and machine learning in the precision health management field, focusing on the value of big health data (i.e., big data in health care). We begin by exploring wearable devices as critical tools for gathering extensive health information, followed by an in-depth discussion of how nanosensors enhance data quality. Subsequently, we highlight the contributions of machine learning algorithms to the precise analysis of big health data and propose several proactive health management strategies from the perspective of “diagnosis-analysis-prevention”. Finally, we present perspectives on the future integration of these technologies to advance comprehensive health management, precision diagnostics, and personalized medicine for older individuals.

## Graphical abstract

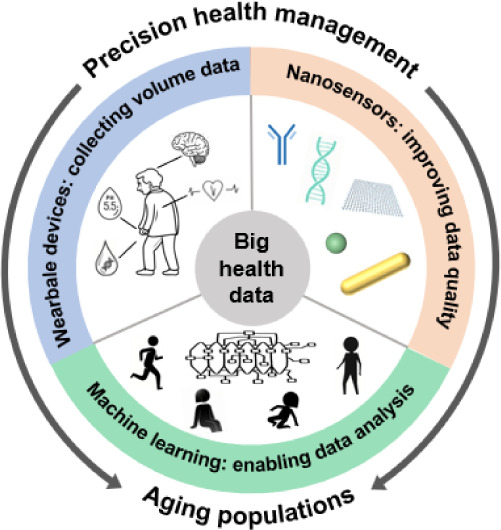

## Introduction

The rapidly growing life expectancy has presented significant health challenges and substantial socioeconomic burdens worldwide.^1^ As people age, they typically face higher risks of chronic diseases, such as Alzheimer’s disease, diabetes, arthritis, and cardiovascular conditions.^2,3^ The morbidity and severity of such diseases are highly influenced by the interplay between genetics, lifestyle, and environmental factors, leading to considerable variability among individuals and necessitating the precise management of these conditions.^4^ However, this type of management requires long-term monitoring and care, which imposes intense pressure on healthcare systems because of the huge personnel demands and economic costs.^5^ For this reason, a primary societal goal is to develop precision health management systems that can automatically monitor aging-related health issues at the individual level whenever and wherever possible. Such systems can continuously collect health data from individuals and provide timely support for decision-making.^6^ As a result, proactive approaches can be implemented to reduce diagnostic and hospitalization costs and shorten waiting times for consultations, thereby significantly improving healthcare efficiency.^7^ Therefore, the development of precision health management systems offers a powerful solution for alleviating the socioeconomic burden associated with caring for older adult populations.

Rapid and ongoing advances in wearable technologies have recently inundated the healthcare sector with vast amounts of health data for precision health management, benefiting older populations.^8^ Wearable devices offer several advantages for data collection, including diverse data sources, a variety of data types, and an extended data collection period,^9–11^ which make them powerful tools for monitoring health status and diagnosing diseases in older adults. For instance, continuous and real-time monitoring of physical and emotional states through wearable devices can help identify falls, strokes, and seizure disorders. Additionally, biophysical and biochemical information, such as heart rate, blood pressure, glucose levels, and biomarkers, can be captured by these devices and used for diagnosis and intervention when neurodegenerative diseases, cardiovascular conditions, diabetes, and other health disorders are present. Because of their excellent performance in the collection of big health data, wearable devices, combined with machine learning-based analytics techniques, have driven a revolutionary shift from hospital-centered to human-centered precision healthcare for older adult populations.^12^

Older adults have distinct and complex needs for precision health management because of the pathological and physiological changes that occur with aging. For instance, older adults often suffer from multiple coexisting diseases, requiring the simultaneous monitoring of various biomarkers.^13^ Moreover, as they age, their physiological and biochemical indicators undergo specific changes, and even subtle variations in these changes can signal the onset of disease, making the sensitive detection and interpretation of these signals essential.^14^ Consequently, while wearable devices enable the collection of vast amounts of health data, researchers have devoted considerable effort to enhancing the quality of this data. For this reason, nanosensors, offering excellent sensitivity and specificity, have attracted considerable attention for precision health care benefiting older individuals.^15^ Key components of nanosensors are typically nanomaterials, and the intrinsic properties of these nanomaterials, including ultrafine size, large surface-to-volume ratio, and diverse physical characteristics, allow nanosensors to produce remarkable signal responses to weak physical and biochemical stimuli, thereby enhancing sensitivity in detecting health parameters.^16^ Additionally, nanosensors can be functionalized with recognition molecules that selectively bind to target biological and chemical analytes, further increasing their specificity for detecting biochemical indicators.^17^ Enhanced by these features, nanosensors have been integrated into wearable devices to improve the veracity and accuracy of health data collected for precisely managing the care of older individuals.

Thus, nanosensor-enhanced wearable devices integrated with machine learning, which rely on big health data, are critical for precisely managing the health care of older adult populations (Scheme 1). Herein, we discuss this topic. First, we introduce various types of wearable devices as critical sources for collecting massive amounts of health data. Second, we highlight nanosensors with a deep discussion on how they improve the performance of wearable devices and enhance the quality of health data. Third, we delve into data analytics for managing the health care of older adult populations, with particular emphasis on machine learning. Then, we propose several proactive health management strategies based on data analytics for older adults, from the perspective of “diagnosis-analysis-prevention”. Lastly, we address the challenges associated with translating nanosensor-integrated wearable devices and machine learning into daily use and outline future perspectives regarding nanosensor-integrated wearable devices in precision health care management for older adult populations.

**Scheme 1:**
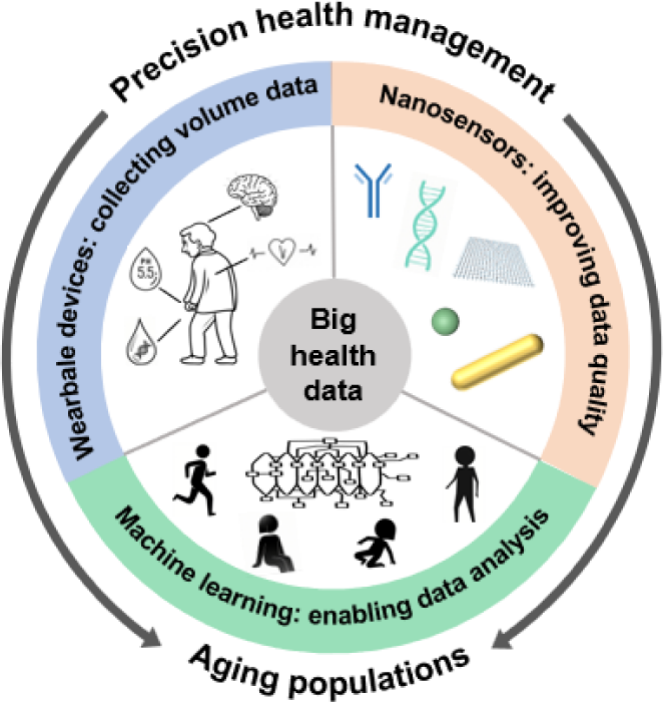
Schematic Showing that Precision Health Management for Older Adult Populations—Involving Collecting Volume Data, Improving Data Quality, and Enabling Accurate Data Analysis—Relies on the Synergy of Wearable Devices,Nanosensors, and Machine Learning, Which in Turn Rely on Big Health Data^a^

a Wearable devices collect aging-related physiological and biochemical data. Nanosensors enhance the sensitivity and selectivity of the aging-related data collected by wearable devices. Machine learning enables the accurate analysis of aging-related health data.

## Wearable Devices as Important Sources of Aging-Related Health Data

Aging is typically accompanied by gradual and progressive changes at the molecular, cellular, tissue, and organ levels, which, in turn, affect the function of various systems.^18,19^ Monitoring data associated with these physiological and pathological changes is crucial for precision health management in older adult populations. Wearable devices provide real-time, multifaceted information about an individual’s health condition, making them essential tools for effective health management. To date, a diverse range of wearable devices has been developed, including watches, bracelets, rings, glasses, fabrics, patches, headphones, and more.^20,21^ These “smart” devices are equipped with multimodal sensing elements, such as thermometers, sphygmomanometers, accelerometers, optical sensors, and electrical sensors, enabling continuous and noninvasive monitoring of various biophysical and biochemical indicators.^22,23^ Consequently, vast amounts of health data are collected from different sources, including skin, sweat, tears, saliva, and urine, facilitating the advancement of precision health management for the older adult population^24–26^ (Figure 1). In this section, we present different types of health data collected by wearable devices, namely biophysical, biochemical, environmental, and lifestyle data.

**Figure 1:**
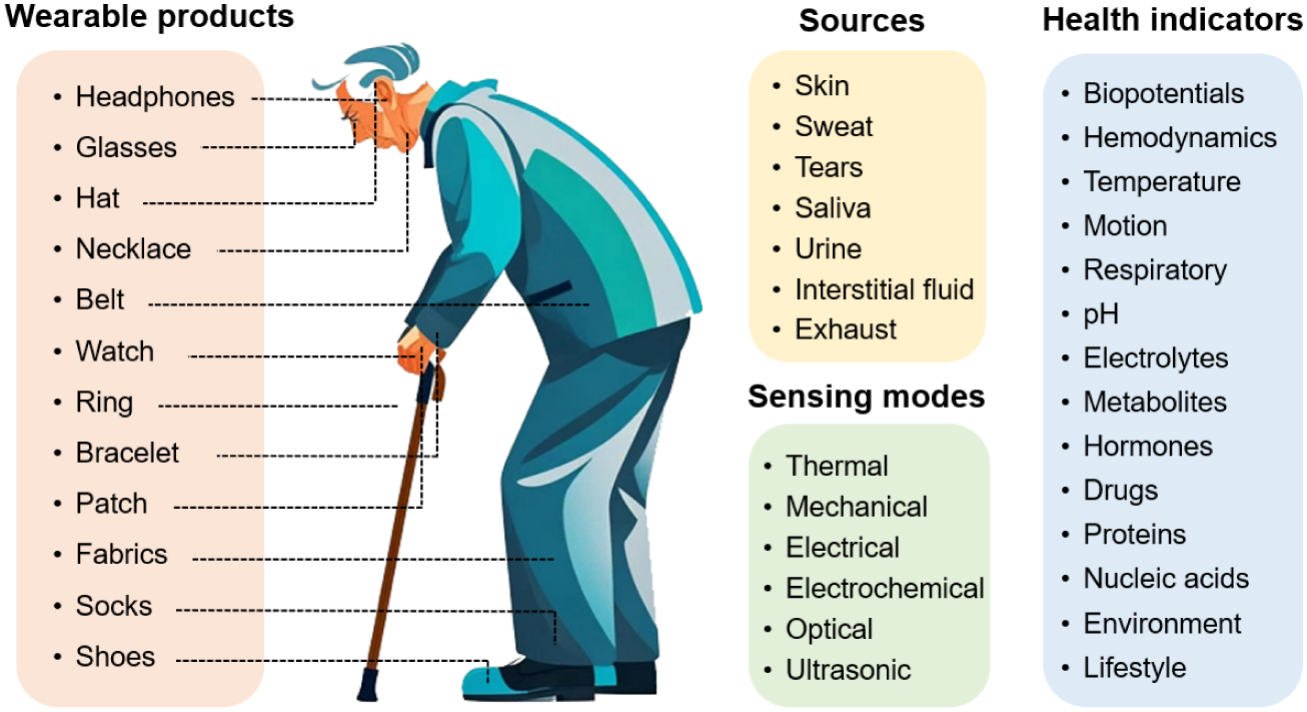
Health monitoring of older individuals can be facilitated by various wearable devices, which collect health data from different sources via different sensing modes to reflect different health indicators.

### Biophysical Data

#### Electrophysiological Data

Electrophysiological signals originate from the flow of ion currents in specific organs and are closely associated with many physiological processes.^27^ It is well recognized that electrophysiological parameters gradually and progressively change during aging and are highly related to the deterioration of organ function in older adults.^28^ Thus, obtaining electrophysiological information from older adults is important, and wearable devices offer a noninvasive and continuous strategy for this purpose. Routinely, the main electrophysiological data are generated by the heart, as recorded in an electrocardiogram (ECG); the brain, in an electroencephalogram (EEG); and the muscles, in an electromyogram (EMG).^29^ Specifically, an ECG, a recording of the electrical activity from cardiac muscles, is clinically used to identify minute heartbeat changes. EEG data, reflecting the spontaneous and rhythmic discharge activity of neurons beneath the scalp, have been regarded as important indicators for diagnosing neurological disorders, including Alzheimer’s disease, Parkinson’s disease, epilepsy, and stroke. EMG data are used to measure the response of the muscle to electrical stimulation of the nerves and thus are clinically applied for the monitoring of neuromuscular behaviors and disorders. In addition, other bioelectrical recordings, such as an electrooculogram (EOG), an electroretinogram (ERG), an electrogastrogram (EGM), and a galvanic skin response, also provide valuable information for the assessment of health status and disease diagnosis. For example, Wang et al. developed a skin sensor based on a nanomesh-reinforced, gas-permeable hydrogel to enable the continuous monitoring of electrophysiological signals (Figure 2A).^30^ Such a sensor was successfully used to make various recordings, including ECG, EEG, EMG, EOG, motor conduction velocity (MCV), auditory brainstem response (ABR), and visual evoked potential (VEP) for a period as long as 8 days, offering a powerful routine for timely diagnosis and precise management of cardiac diseases and neurological disorders in older adult populations.

**Figure 2:**
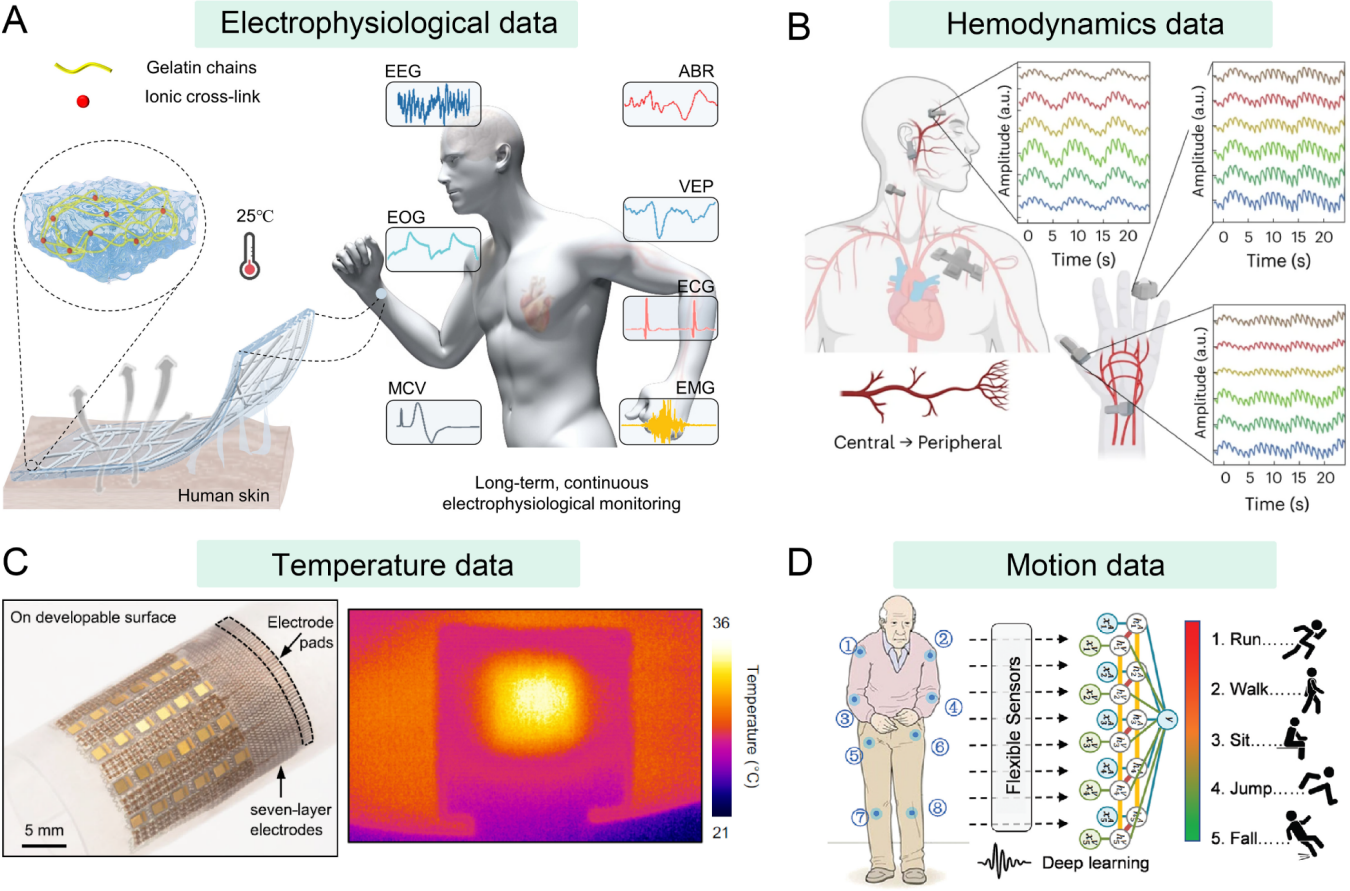
Wearable devices for collecting biophysical data in older adults. (A) Wearable skin sensor based on a nanomesh-reinforced, gas-permeable hydrogel for the monitoring of electrophysiological data. Reproduced with permission from ref. 30. Copyright 2024 AAAS. (B) A synchronized wearable system for the monitoring of hemodynamics data. Reproduced with permission from ref. 35. Copyright 2023 Springer Nature. (C) A wearable photoacoustic patch for the imaging of core temperature. Reproduced with permission under Creative Commons Attribution 4.0 International License from ref. 43. Copyright 2022, The Authors. (D) A wearable mechanical system for the collection of motion data. Reproduced with permission from ref. 54. Copyright 2022 Wiley-VCH.

#### Hemodynamics Data

Aging is associated with a progressive decline in the structure and function of blood vessels and the heart, accompanied by increased thickness and stiffness of the larger arteries, resulting in decreased compliance.^31^ Therefore, older adults face a higher risk of cardiovascular issues than younger adults. Hemodynamic parameters such as heart rate, blood pressure, and blood oxygen saturation have been well-recognized as indicators of the status of blood vessels and the heart.^32^ Heart rate, defined as the number of heartbeats per minute, indicates the heart rhythm. An abnormal heart rate or sudden changes in heart rate may suggest irregular heart function. Blood pressure, presented as systolic and diastolic pressure values, is used to reflect the force exerted by blood against the artery wall. Alterations in blood pressure may cause serious issues such as stroke, heart failure, and even heart attack, and it is worth mentioning that the risk of hypertension significantly increases with age.^33^ Another important hemodynamic parameter, blood oxygen saturation, is closely related to the function of breathing and heartbeat. Monitoring this hemodynamic information is crucial for evaluating circulatory function and diagnosing cardiovascular diseases such as atherosclerosis, hypertension, and arrhythmia.^34^ To date, wearable devices have been used for noninvasive and continuous measurement of this information in older adults. Rogers et al. developed a synchronized wearable system by combining ECG and multispectral photoplethysmography for the continuous monitoring of hemodynamic data associated with vascular resistance, cardiac output, and blood pressure (Figure 2B).^35^ With the assistance of a machine learning algorithm (support vector machine), these data were used to identify different hemodynamic states, namely, the state of healthy individuals, individuals with hypertension undergoing hemodynamic stimuli, or patients after cardiac surgery, holding great promise for precisely managing the health of older adults suffering from cardiovascular diseases. In addition, Xu’s group developed several ultrasound-based wearable systems to monitor the hemodynamics in deep tissues, considering that many chronic diseases originate or manifest in these regions.^36,37^ In 2021, they developed a stretchable ultrasonic phased array to detect the hemodynamic signals in tissues as deep as 14 cm.^36^ Using this array, they collected data in real time, including the Doppler spectra of cardiac tissues and central blood flow waveforms. In subsequent work, they constructed a fully integrated ultrasound system by interfacing wearable sensors with a miniaturized control circuit, a signal preconditioning unit, and a wireless data communication module.^37^ Hemodynamic signals, including blood pressure, heart rate, and cardiac output, were continuously recorded and then analyzed via a machine learning algorithm. The system was able to continuously track signals from a mobile individual for more than 12 h, providing a robust platform for the health monitoring of cardiac rehabilitation patients and other populations with high cardiovascular risk.

#### Body Temperature

Body temperature is a vital sign modulated by the thermoregulatory and immune systems, which become less physiologically efficient with aging.^38^ Body temperature can be measured as core body temperature (e.g., oral, axillary, or rectal) or skin temperature (e.g., at the wrist or lateral rib cage). Due to less efficient thermoregulatory responses and diminished heat-producing body mass, older adults usually have lower core body temperatures than younger adults.^39^ Moreover, older adults are more vulnerable to heat or cold stressors, and abnormal changes in their body temperature are associated with microbial infection, inflammation, and neurodegeneration.^40^ Therefore, body temperature monitoring provides valuable information, helping to evaluate the health of older adults and predict disease. Typical temperature sensors, such as platinum resistance thermometers, thermistors, thermocouples, and temperature-integrated circuits, have been incorporated into wearable devices for monitoring the health of older adults.^41^ Recently, with advances in synthetic chemistry, skin-friendly and flexible materials with temperature-responsive properties have been fabricated for precise monitoring of skin and core temperature.^42,43^ For example, Zhang et al. fabricated a wearable temperature sensor based on thermoplastic polyurethane fiber.^42^ The sensor exhibited broad linearity from 20 to 40 °C with a high resolution of 0.2 °C and satisfactorily monitored temperature changes during exercise, demonstrating significant utility for remotely monitoring the health of older adults while they are at home. In another work, Xu and collaborators designed a wearable photoacoustic patch for imaging hemoglobin and core temperature with high precision and a fast response time (Figure 2C).^43^ In this patch, a pulsed laser excited hemoglobin to generate acoustic waves, and the signal amplitude was linearly related to core temperature in the range of 10 to 55 °C. These wearable devices for temperature data collection provide efficient tools for evaluating the health of older adults, enabling timely diagnosis of fever, inflammation, or infection.

#### Motion Data

The daily activities of older adults, such as postures, sleep, hand gestures, and facial motions, provide important information for health care management.^44–46^ For example, analyzing gait and posture characteristics via wearable devices offers an efficient means of identifying whether a person has fallen—important, since falls due to abnormal health or accidental conditions are responsible for more than 680,000 deaths per year.^44^ In addition, postures, hand gestures, facial expressions, and movements associated with speech have been reported as useful features for assessing the progression of neurodegenerative diseases.^45^ Wearable devices have been used to detect these motions in older adults, collecting detailed information including gait parameters (e.g., speed, cadence, step length, weight distribution),^47^ postures (e.g., walking, standing, lying, sitting, climbing stairs, swimming, running, jumping),^48,49^ facial motions (e.g., elevating eyebrows, closing eyes, smiling, smiling while baring teeth, frowning),^50,51^ facial expressions (e.g., happiness, surprise, sadness, anger, disgust),^52^ and hand gestures (e.g., extension, flexion, adduction, and abduction of an individual finger or multiple fingers).^53^ This is achieved by placing wearable devices on diverse body parts, including the ear, lip, chin, neck, chest, arm, wrist, back, hip, thigh, shank, knee, ankle, and foot, to collect raw data, which are processed by machine learning algorithms. Huang and collaborators fabricated ultrarobust fibrous mechanical sensors to construct a wearable system for the monitoring of muscle movement, body tremors, wrist pulses, respiration, and gestures. Importantly, this system, integrated with a deep learning algorithm, could successfully recognize different body postures, including running, walking, sitting, jumping, and falling, displaying significant potential for monitoring the health of older adults who live alone (Figure 2D).^54^ Hausdorff et al. used a wearable lower back inertial device to collect motion data, which were processed by machine learning models to estimate the step length in older adults and even in patients with Parkinson’s disease.^55^ In another work, Jia et al. reported the development of an EMG sensor based on flexible microneedle electrode arrays for the monitoring of facial palsy, which offers a powerful strategy for the diagnosis and management of neurological disorders in older adult populations.^50^ Thus, wearable devices provide a highly accessible way to obtain motion data that reflect rich health information, and they are expected to play an important role in precision health management for older adult populations.

In addition to the aforementioned biophysical signals, wearable devices are also used to monitor signals such as respiration^56,57^ and mechanical acoustics.^58,59^ Wearable devices are capable of monitoring multiple biophysical data types to enhance the accuracy of health assessments and disease diagnoses. Generally speaking, biophysical data collected by wearable devices can provide real-time, dynamic physiological feedback and information about health changes to quickly and directly evaluate the immediate health risks of older adults. This is particularly significant for dynamic health management and early warning of acute conditions such as heart failure, stroke, and falls.

### Biochemical Data

#### pH

The pH indicates the molar concentration of hydrogen ions in a solution and is an important biochemical parameter for humans. Physiologically, pH is subtly modulated by acid–base homeostasis and varies from 1 to 8 in different organs.^60^ For example, the pH of blood is typically 7.35 to 7.45, while the pH of the stratum corneum is physiologically 4.1 to 5.8. An abnormal pH in specific tissues and organs may significantly affect enzyme activity, leading to metabolic imbalance and even severe diseases. This may occur in older adults, as, in comparison to younger adults, they typically exhibit reduced regulation of metabolic homeostasis and face a higher risk of damage associated with alkalinity or acidity.^61^ Therefore, pH can serve as an efficient indicator of the health of specific organs and tissues. As an example, increased skin pH is associated with inflammatory skin diseases and epidermal conditions.^60^ Also, decreased urine pH is related to insulin resistance in the renal tubules.^61^ Therefore, it is of great importance to continuously monitor the pH of specific tissues to manage the health care of older adult populations. Wearable devices containing pH sensors have been developed to measure the pH of sweat, urine, tears, interstitial fluid, or saliva. The most common pH sensors are based on the ion-selective electrode, the sensitivity of which is limited to about 59 mV per pH unit.^62^ Recently, Takei’s group developed a flexible, charge-coupled device (CCD)-based wearable sensor for pH monitoring of sweat (Figure 3A).^62^ Because of its excellent electron charge transfer, this sensor displays a sensitivity of 240 mV per pH unit, showing great potential for precise monitoring of the sweat pH of older adults. In addition, Park and collaborators developed a wearable colorimetric pH sensor and integrated it into clothing for the measurement of sweat pH, allowing for the continuous monitoring of cystic fibrosis in older adults.^63^ These pH-monitoring wearable devices offer an efficient means of reflecting acid–base imbalance in older individuals with diabetes and kidney disease, as well as assessing their nutritional status.

**Figure 3:**
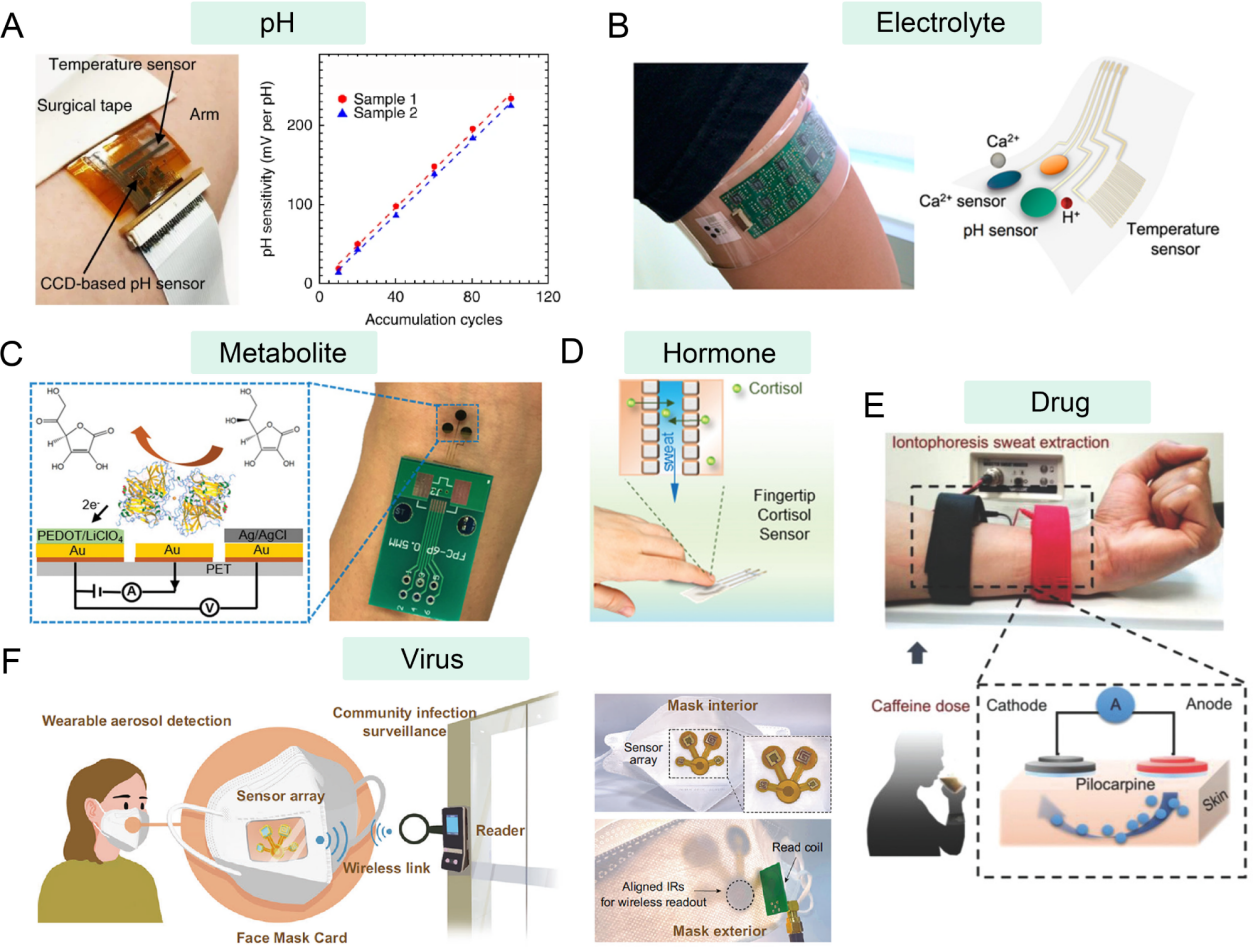
Wearable devices for the collection of biochemical data in older adults. (A) Flexible, charge-coupled device-based wearable sensor for pH monitoring in sweat. Reproduced with permission from ref. 62. Copyright 2018 Springer Nature. (B) A wearable electrochemical device for the continuous monitoring of Ca^2+^ in biofluids. Reproduced with permission from ref. 66. Copyright 2016 American Chemical Society. (C) A wearable electrochemical sensor to monitor vitamin C. PEDOT refers to poly­(3,4-ethylenedioxythiophene) and PET refers to poly­(ethylene terephthalate). Reproduced with permission from ref. 72. Copyright 2020 Wiley-VCH. (D) A touch-based, noninvasive electrochemical sensor for rapid and reliable detection of hormone levels in fingertip sweat. Reproduced with permission from ref. 76. Copyright 2021 Wiley-VCH. (E) A wearable electrochemical sensor for the monitoring of the methylxanthine drug caffeine. Reproduced with permission from ref. 80. Copyright 2018 Wiley-VCH. (F) A wearable face mask sensor for the rapid and on-site detection of respiratory virus aerosols. Reproduced with permission under Creative Commons Attribution 4.0 International License from ref. 85. Copyright 2024, The Authors.

#### Electrolytes

Electrolyte homeostasis is a precondition for various life processes, and electrolyte imbalance is associated with irregular body function, disrupted homeostasis, and an increased risk of life-threatening complications.^64^ Aging reduces the level of electrolytes and decreases the percentage of water in the human body, making the older adult population more vulnerable to electrolyte imbalance.^64^ It has been well recognized that monitoring changes in electrolytes (e.g., Ca^2+^, Na^+^, K^+^, Mg^2+^, Cl^–^, PO_4_^3–^) provides helpful information about exercise activity and hydration state and can help diagnose diseases such as hypertension, chronic renal failure, and hyperkalemia.^65^ To date, ion-selective electrodes have been integrated into wearable devices for monitoring electrolytes in various samples. For example, Javey et al. designed a wearable electrochemical device for the continuous monitoring of Ca^2+^ in biofluids by interfacing a flexible Ca^2+^ sensor with a printed circuit (Figure 3B).^66^ This device enabled the measurement of Ca^2+^ in sweat, urine, and tear samples and can be applied for diagnosing diseases such as kidney stones and osteoporosis. In another work, Haick et al. constructed a microneedle-based gate transistor sensor for the monitoring of Na^+^ in interstitial fluids.^67^ They integrated this sensor with a wireless data transmitter and the cloud for data processing and analysis, making it convenient and practical for precision health management, benefiting older adults.

#### Metabolites

The relationship between aging and metabolism has long been investigated. Early studies showed that the rate of metabolism and the products of metabolism (i.e., metabolites) play an important role in the development of aging processes,^68^ and dysregulated metabolism has been regarded as a hallmark of aging. Moreover, aging-related dysregulated metabolism is tightly associated with several diseases; for example, dysregulated glucose metabolism is associated with diabetes, and dysregulated lipid metabolism is linked to cardiovascular diseases.^69^ Therefore, measuring the concentrations of metabolites is greatly important for monitoring the health status of older adults and for diagnosing aging-related diseases. To date, wearable devices have been used to monitor metabolites and nutrients, including glucose, lactate, vitamin C, volatile organic compounds, and amino acids in older people.^70–73^ Among the metabolites, glucose and lactate in sweat and urine have been widely monitored by wearable patches and fingertip microgrids through oxidase-catalyzed electrochemical reactions.^70,71^ Vitamin C has been specifically and continuously measured in biofluids such as sweat, urine, and blood by a wearable electrochemical sensor, which offers an efficient strategy to monitor vitamin C levels and helps evaluate nutritional status for precisely managing the nutrition of older adults (Figure 3C).^72^ Besides, amino acids have been monitored by a molecular-imprinted polymer (MIP)-based wearable electrochemical sensor for assessing the intake of amino acids and evaluating the risk of metabolic dysregulation.^73^ Generally, wearable devices provide a powerful tool for collecting metabolic data for the precise management of nutritional status and metabolic diseases in older individuals.

#### Hormones

During the aging process, the secretion of hormones by the hypothalamic-pituitary axis undergoes significant changes, which subsequently impact the release of terminal hormones.^74^ Additionally, chronic diseases, inflammation, and poor nutritional status associated with aging can also influence hormone secretion. The resulting alterations in hormone levels can contribute to the loss of bone, muscle mass, and strength in older adults, leading to a decline in physical function and an increased risk of disease.^75^ Therefore, assessing hormone levels in older individuals is essential for monitoring their overall bodily functions and for the prevention of endocrine disorders and related metabolic diseases. For this purpose, Wang’s group constructed a touch-based, noninvasive electrochemical sensor for the rapid and reliable detection of hormone levels in fingertip sweat (Figure 3D).^76^ Such a sensor specifically recognizes cortisol and generates electrical signals for rapid and reliable detection. Additionally, Cheng et al. reported a wearable electrochemical patch for the simultaneous detection of 2 adrenocortical stress markers, cortisol and dehydroepiandrosterone (DHEA), in sweat.^77^ This multiplexed sensor exhibited a fast response to cortisol and DHEA within 5 min, as well as high sensitivity, with a limit of detection (LOD) of 115 pM for cortisol and 390 pM for DHEA. When integrated with a wireless module, it was used for ambulatory detection. This work provides a rapid and sensitive tool for the detection of hormones, enabling endocrine monitoring for precision health management benefiting older adults.

#### Drugs

Compared with younger adults, older adults face a higher risk of illness and greater reliance on medications. Older adults must often take multiple medications simultaneously, which can considerably amplify the potential for side effects.^78^ Besides, the nature of side effects can also change as aging affects the absorption, distribution, metabolism, and excretion of drugs in the body, leading to different pharmacological responses and side effects compared to those in younger adults. Furthermore, older individuals show substantial variability in the metabolism of drugs.^79^ For all of these reasons, regular drug monitoring is vital for older adults to ensure safe and effective medication use, manage complex health needs, prevent adverse effects, and enhance overall well-being. For regular drug monitoring, wearable devices offer an alternative to conventional drug tests, which rely on invasive blood draws. Javey et al. designed a wearable device based on an electrochemical sensor for the monitoring of the methylxanthine drug caffeine (Figure 3E).^80^ This device was used to monitor caffeine levels in sweat under different conditions, including varying drug doses, as well as different measurement times after drug intake. This work represents an efficient strategy for drug monitoring benefiting older adults. In another work, Zhang’s group developed a wearable plasmonic sensor for quantifying acetaminophen in sweat to reflect an individual’s drug metabolism.^81^ Owing to its advantages in sweat collection, a flexible microfluidic chip was integrated with the plasmonic sensor to enable rapid sweat collection. Such an integrated device can be placed on various body positions, offering an efficient tool to noninvasively monitor the acetaminophen level in sweat for drug pharmacokinetics. Because of their ability to continuously monitor medication, wearable devices will play an indispensable role in the precise administration of daily medications and personalized treatment for older individuals.

#### Other Biomarkers

Nucleic acids and proteins, 2 important biomarkers, have been widely used for the diagnosis of various diseases, such as genetic disorders, infections, and cancers.^82,83^ Wearable devices offer a robust tool to monitor nucleic acid and protein biomarkers for the precise diagnosis and management of diseases affecting older adults. Collins et al. integrated lysis agents, reverse transcription recombinase polymerase amplification, CRISPR, and lateral flow assays into a wearable face mask for the noninvasive measurement of pathogen-derived nucleic acids.^84^ Using this sensing system, the researchers detected severe acute respiratory syndrome coronavirus 2 (SARS-CoV-2) via colorimetric results at room temperature within 90 min, enabling the rapid and sensitive detection of pathogens. This wearable system has significant application prospects for the rapid screening and daily monitoring of infectious diseases in older adult populations. In addition to DNA molecules, proteins and virus particles can also be detected by wearable sensors.^85,86^ Liu et al. developed a wearable face mask sensor for the rapid and on-site detection of respiratory virus aerosols (Figure 3F).^85^ In their design, antibodies were used to capture and recognize the virus antigen, and then electrical signals were generated and amplified by a responsive hydrogel-modulated resonant sensor. Moreover, this wearable sensor achieved the simultaneous detection of multiple respiratory viruses, including SARS-CoV-2, influenza A H1N1 virus, and respiratory syncytial virus, indicating great promise for precise disease diagnosis. Considering the indicative role of biomarkers in diseases, wearable devices for nucleic acid and protein detection have significant potential for applications such as screening at-risk older adults, enabling early disease diagnosis, and monitoring for disease recurrence.

Solid-state epidermal biomarkers have recently attracted extensive attention in continuous health monitoring because of their ubiquitous and easy-to-access properties.^87^ It has been reported that solid-state cholesterol can serve as an indicator of hyperlipoproteinemia; solid-state lactate, as an indicator of cardiovascular disease; and solid-state glucose, as an indicator of diabetes.^87^ Liu and collaborators designed a wearable sensor based on an ionic-electronic bilayer hydrogel for the monitoring of solid-state biomarkers, including water-soluble lactate and water-insoluble cholesterol.^87^ This bilayer hydrogel allowed for the sequential dissolution and electrochemical detection of solid-state analytes, and the concentrations measured by the wearable sensor were highly correlated with the biomarker concentrations in blood. This work extended the range of biomarkers beyond solution-state analytes and opened up new avenues for health monitoring and disease diagnosis, ones not requiring biofluid acquisition.

Compared to biophysical indicators, biochemical indicators collected by wearable devices focus more on reflecting the health information of older adults at the molecular and cellular levels. They provide a more comprehensive view of the metabolic status and functioning of internal organs and are thus advantageous for revealing the risk of chronic diseases. Additionally, these biochemical indicators can reflect health status over a longer period, making them particularly suitable for chronic disease prevention and management. When used for comprehensively assessing the health status of older adults, wearable devices should be designed to collect both types of data to more effectively identify health risks and formulate personalized intervention measures.

### Environmental and Lifestyle Data

At the subclinical level, aging is characterized by a decline in function across various systems, such as the motor, nervous, and immune systems.^88^ However, the rate of these changes varies significantly among individuals.^89^ For instance, some people may experience illness and frailty in their sixties, while others maintain good health and vitality into their nineties. Notably, these differences are not primarily due to significant genetic variation but rather to environmental factors and lifestyle choices that influence health status and the aging process.^89^ Environmental influences encompass diverse exposures, such as chemical, physical, natural, biological, and social factors.^90^ For example, harmful chemicals in air and water can lead to toxicity and illness in older adults. Biological agents, such as viruses and bacteria, can cause infections, and a noisy social environment may contribute to insomnia. Lifestyle choices, including diet, sleep patterns, physical exercise, smoking, and alcohol consumption, are also well-known to play a critical role in overall health. Therefore, collecting and monitoring data on these environmental and lifestyle factors is essential for effectively and precisely managing the health of older adults. Wearable devices, directly worn on the human body, can monitor real-time environmental data near the human body and record lifestyle habits, providing users with a large amount of data and information to precisely manage their health. For instance, Lee et al. developed a flexible chemiresistive membrane sensor for the ultrasensitive detection of formaldehyde.^91^ Their membrane, composed of a zeolitic imidazole framework and polymer-coated TiO_2_ films, removes ethanol interference to achieve the ultrahigh-specificity detection of formaldehyde. Because of the excellent response capacity of the TiO_2_ films, the sensor displays high sensitivity, with a response (resistance ratio of more than 1100) to 5 ppm formaldehyde. Beyond environmental data, plenty of wearable devices, including commercial watches, have been utilized to record lifestyle information like exercise and sleep data.^92^ The collected data are then analyzed by an algorithm to provide older adults with personalized advice on daily health management.

Biophysical, biochemical, environmental, and lifestyle data collectively indicate the health status of older people and reveal health disparities among them. Integration of these data helps provide a comprehensive assessment and prediction of the health condition of older adults, supporting precision health management. As wearable devices monitor an increasing number of health-related indicators and the volume of collected data grows, their assessment and prediction of health status will become even more accurate, allowing them to play an even greater role in the health management of older adults.

## Nanosensor-Enhanced Wearable Devices for Improving Quality of Aging-Related Health Data

Aging is associated with a decline in organ function, which affects the health status of older adults in multiple ways, making them more susceptible to illness.^93^ Even slight variations in physiological parameters and minimal changes in biochemical indicators can trigger health issues in older adults, leading to the onset of diseases.^94^ Therefore, monitoring subtle changes in physiological signals and minute variations in biomarker concentrations is invaluable for precision health management and disease diagnosis, benefiting older populations. However, the extreme weakness of these physiological and biomarker signals, combined with their susceptibility to interference from environmental factors and biological matrices, poses a significant challenge to their accurate detection.^95–97^ To address this challenge, developers of wearable devices for older adults often use sensors based on microelectromechanical systems (MEMS). For example, Chen’s group reported micro Inertial Measurement Units (μIMUs)-based MEMS sensors to monitor the sit-to-stand motion of older adults for multi risk-level sarcopenia-prone screening, which achieved high accuracy (98.32%) in classifying healthy versus sarcopenia-prone individuals.^98^ In another work, they integrated Internet-of-Things technology and μIMUs sensors to monitor daily gait activity in older adults, which exhibited high accuracy (97.41%) in identifying sarcopenia-prone individuals.^99^ Although MEMS sensors are miniaturized, their sensitivity is often constrained by their micrometer-scale dimensions, particularly when detecting extremely low concentrations or subtle changes. Moreover, MEMS sensors operate primarily by physical detection principles and require surface modifications to impart specific biological and chemical functionalities when they are used to detect biological and chemical parameters. However, the limited specific surface area of MEMS sensors restricts both the efficiency of surface functionalization and the specificity of target recognition, thereby impeding further enhancement of their detection performance in biological and chemical sensing.

The limitations of MEMS sensors can be overcome using nanosensors. Nanosensors capitalize on the small size—but large specific surface area—of nanomaterials, which enable these sensors to produce substantial signal responses to specific physical or biochemical stimuli, resulting in high detection sensitivity.^15,16^ Additionally, nanosensors possess adjustable signal response mechanisms, which can enhance specificity toward stimuli.^15,100^ These sensors can also be modified with highly specific recognition molecules, allowing for the selective identification of biochemical analytes and achieving targeted detection.^17^ Thus, integrating wearable devices with nanosensors can significantly improve device sensitivity and specificity, thereby ensuring the accuracy of data collection and facilitating precision health management for older adults.

Generally, nanosensors are composed of 2 principal functional components: a sensing recognition unit and a signal transduction unit.^15^ The sensing recognition unit is primarily responsible for selectively binding to or interacting with the target parameter, which induces physical or chemical changes in the recognition unit.^16^ The signal transduction unit translates these changes into a quantifiable output signal, the intensity of which is directly proportional to the concentration or strength of the target parameter, thereby enabling its detection.^101^ Both the sensing recognition and signal transduction units rely on nanomaterials. In the sensing recognition unit, nanomaterials offer a large specific surface area and readily tunable surface chemistry, facilitating the precise modulation of the nanosensor’s selectivity for the target. Furthermore, nanomaterials can be functionalized with a multitude of biorecognition elements, such as antibodies, nucleic acids, or MIPs, which exhibit highly specific binding or reactivity with targets, thus ensuring stringent detection specificity.^16^ In the signal transduction unit, the inherent properties of different nanomaterials dictate variations in transduction mechanisms, which include optical (e.g., fluorescence, colorimetric, Raman), electrochemical, magnetic, and mechanical mechanisms.^102–105^ Optical and electrochemical mechanisms are particularly prevalent in wearable devices because they do not rely on complex detection equipment. Owing to the significant specific surface area and quantum size effects characteristic of nanomaterials, even minute interactions can elicit substantial signal changes, affording nanosensors a notable advantage in enhancing detection sensitivity. This chapter will therefore focus on the contribution of nanosensors to wearable devices, discussing how these sensors improve their sensitivity (summarized in Table 1) and their specificity (summarized in Table 2).

**Table 1:** Improving the Sensitivity of Wearable Devices with Nanosensors

Mechanism for enhanced sensitivity	Parameters detected	Sensing mode	Nanomaterials	LOD or sensitivity	Ref.
Enhanced signal transduction by high-performance nanomaterials	Cytokines	Electrical	Graphene	LOD: 880 fM	^101^
	Motion and temperature	Electrical	Carbon nanotube	LOD of strain: 1%	^106^
	EOG	Electrical	Carbon quantum dots	/	^107^
	Pressure	Electrical	MXene	Sensitivity: 298.4 kPa^–1^ for 1.4–15.7 kPa; 171.9 kPa^–1^ for 15.7–39.3 kPa	^102^
	Temperature	Piezoresistive	Doped silicon	Temperature coefficient of resistance: – 37270.72 ppm °C^1–^	^110^
	Temperature	SERS	Gold nanoparticles	Temperature-sensing resolution: 0.2 °C	^103^
	Drug	SERS	Silver nanocubes	LOD: 0.01 nM	^104^
Enhanced signal transduction by responsive structures	Pressure	Electrical	Kirigami nanowire	Sensitivity: 35.2 mV Pa^–1^	^113^
	Pressure	Electrical	Tip array-based bipyramidal microstructure	Sensitivity: 8.5 V kPa^–1^ LOD: 0.14 Pa	^114^
Enhanced nanomaterial-target interaction	Cortisol	Fluorescent	MOFs	LOD: 10^–9^ M	^117^
	Humidity	Electrical	COFs	/	^115^
	Na^+^	Electrochemical	Mesoporous graphene	Sensitivity: 65.1 ± 0.25 mV decade^–1^	^118^
	NO_2_	Electrochemical	Reduced graphene oxide–ZnO nanosheet	LOD: 43.5 ppb	^119^

**Table 2:** Improving the Specificity of Wearable Devices by Nanosensors

Mechanism for enhanced specificity	Parameters detected	Sensing mode	Nanomaterials	Interference assays	Ref.
Nanomaterials–analytes interaction	Ascorbate	Electrochemical	Modified MOFs	High selectivity against lactate, uric acid, glucose, ethanol, and cortisol	^123^
	L-lactic acid	Fluorescent/colorimetric	Chiral MOFs	High selectivity against D-lactic acid	^124^
Antibody–analytes interaction	CRP	Electrochemical	Thionine/gold nanoparticles	High selectivity against cortisol, IgG, and TNF-α	^126^
	Exosomes	Electrical	Graphene	/	^127^
	Cortisol	Electrochemical	MOFs	High selectivity against cortisone, corticosterone, L-ascorbic acid, uric acid, creatinine, progesterone, cholesterol	^128^
Aptamer–analytes interaction	Cortisol	Electrical	In_2_O_3_	High selectivity against progesterone, testosterone, and serotonin	^134^
	Oestradiol	Electrochemical	Gold nanoparticles–MXene	High selectivity against estradiol, cortisone, cortisol, progesterone, folic acid, serotonin, tobramycin, kanamycin, glucose, and lactic acid	^135^
MIP–analytes interaction	Cortisol	Electrochemical	Prussian blue	High selectivity against lactic acid, glucose, urea, and ascorbic acid	^138^
	Lactate	Electrochemical	Carbon nanotube fibers	High selectivity against lactic acid, urea, glucose, uric acid, NaCl, and CaCl_2_	^139^

### Enhancing Device Sensitivity

#### Enhanced Signal Transduction

Nanomaterials are the core components of nanosensors. Because of their ultrasmall size, nanomaterials exhibit unique physical properties, enabling them to generate enhanced signal responses to stimuli, thereby improving sensor sensitivity as well as data accuracy.^16^ Recently, nanomaterials with excellent electrical properties have been integrated into nanosensors for wearable devices with enhanced detection sensitivity and improved data veracity for precision health management.^101–105^ For example, graphene, a 2D nanomaterial known for its high carrier mobility and excellent flexibility, has been widely used in the development of highly sensitive wearable sensors. Pan et al. designed a flexible graphene-based biosensor for the sensitive monitoring of cytokine storm biomarkers in biofluids (Figure 4A).^101^ This sensor was built by functionalizing a graphene–Nafion field-effect transistor (FET) with aptamer molecules. Because of the excellent electrical properties of graphene–Nafion, the device achieved sensitive detection of cytokines, with a range from 0.015 to 250 nM and an LOD as low as 740 fM, providing exciting possibilities for the diagnosis of inflammatory diseases and cancers. In addition, other carbon nanomaterials, such as carbon nanotubes,^106^ carbon quantum dots,^107^ and carbonized metal–organic frameworks,^108^ have also been reported for the construction of highly sensitive wearable devices for health management. Another kind of nanomaterial, MXene, with intriguing structural and electrical characteristics, has also garnered much interest in recent years in the realm of high-performance wearable sensors.^102,109^ As an example, Wan and collaborators developed an ultrasensitive pressure sensor based on Ti_3_C_2_T_*x*_ MXene–protein nanocomposites.^102^ In their design, MXene was used to fabricate the sensing layer and also served as the electrode layer. Benefiting from the high conductivity of MXene, the wearable pressure sensor displayed high sensitivity: 298.4 kPa^–1^ over the range of 1.4 to 15.7 kPa and 171.9 kPa^–1^ over the range of 15.7 to 39.3 kPa, showing huge potential for motion detection and disease diagnosis applications. In addition, Zhang et al., considering the high electrical conductivity and robust piezoresistive effect of doped silicon, designed an ultrasensitive epidermal temperature sensor array based on a gold-doped silicon nanomembrane.^110^ Because of the gold doping, the freeze-out region shifted to the intrinsic region in carrier density, and the Fermi energy level of the silicon was modulated after doping. This modulation led to an ultrasensitive response to temperature, suggesting that the array could potentially be used for diagnosing and managing temperature-related diseases in older adults.

**Figure 4:**
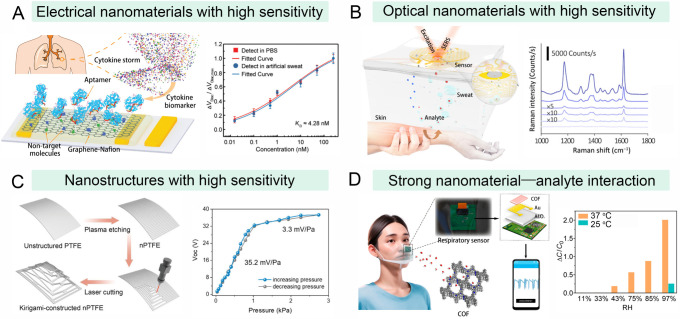
Integration of nanosensors to improve the sensitivity of wearable devices. (A) Flexible graphene-based biosensor for the sensitive monitoring of cytokine storm biomarkers in biofluids. Reproduced with permission from ref. 101. Copyright 2020 Wiley-VCH. (B) An ultrasensitive wearable surface-enhanced Raman spectroscopy (SERS) sensor for the noninvasive detection of trace-amount drugs in biofluids. Reproduced with permission from ref. 104. Copyright 2021 AAAS. (C) A wearable pressure sensor with a kirigami-inspired structure for the sensitive measurement of pulse waves. PTFE refers to poly­(tetrafluoroethylene). Reproduced with permission from ref. 113. Copyright 2022 Wiley-VCH. (D) A covalent organic framework (COF)-based wearable humidity sensor for monitoring the sleep respiratory behavior of sleep apnea patients. RH refers to relative humidity. Reproduced with permission from ref. 117. Copyright 2023 American Chemical Society.

In addition to electrical nanomaterials, optical nanomaterials have been incorporated into nanosensors to improve the sensitivity of wearable devices. In particular, the outstanding plasmonic effects of noble metal nanomaterials offer significant opportunities for surface-enhanced Raman scattering (SERS) and colorimetric detection, achieving high sensitivity.^111,112^ Consequently, such nanomaterials have been extensively exploited for the fabrication of wearable sensors. Ko et al. reported a wearable colorimetric hydrogel patch by incorporating plasmonic gold nanoparticles into a poly­(*N*-isopropylacrylamide) (PNIPAM) microgel.^103^ A temperature change caused a fast phase transition of the PNIPAM microgel and a large volumetric change, leading to a color shift due to the plasmonic coupling effects of the gold nanoparticles. As a result, this wearable patch exhibited a detection range from 29 to 40 °C with a high resolution of 0.2 °C. In another work, Wang and collaborators, considering that Raman signals of analytes can be greatly enhanced by plasmonic nanostructures in close proximity, developed an ultrasensitive wearable SERS sensor based on plasmonic silver nanocubes for the noninvasive detection of trace amounts of drugs in biofluids (Figure 4B).^104^ This SERS sensor was composed of a flexible plasmonic metasurface to act as the sensing component and a flexible electronic system to automatically extract the biofluid samples for real-time tracking of drug variations. Benefiting from the high sensitivity of SERS analysis, the sensor displayed an ultralow LOD of 0.01 nM, which is much lower than that of standard high-performance liquid chromatography (1.1 nM). This work offers a powerful tool to guide older adult patients in personalized medication. Additionally, plasmonic gold nanorods,^111^ silver-based nanovoid arrays,^112^ and gold nanosphere cone arrays^81^ have been employed to develop highly sensitive SERS sensors. These sensors have been integrated with wearable devices for monitoring metabolites and drugs, offering significant potential for the precise management of metabolic diseases in older adult populations.

Another way to enhance signal transduction and improve detection sensitivity is by modulating the structure of nanomaterials, which can be assembled into specific nanostructures or microstructures for this purpose.^113,114^ For example, Chen et al. reported a wearable pressure sensor with a kirigami-inspired structure for the sensitive measurement of pulse waves (Figure 4C).^113^ This kirigami structure was fabricated through the etching and laser cutting of the nanowires. These nanowires enhanced the surface triboelectrification, and the kirigami structure generated an enhanced charge density on the sensing surface, resulting in a superior sensitivity of 35.2 mV Pa^–1^. In another work, Wen et al. designed a tip array structure to improve the sensitivity of wearable pressure sensors.^114^ The tip array-based bipyramidal microstructure, with a high Young’s modulus, increased stress points at the void locations and improved the efficiency of mechanical transfer, enhancing the sensitivity to 8.5 V kPa^–1^ with an LOD as low as 0.14 Pa. Generally, the integration of responsive nanostructures into wearable sensors displays great promise for advancing the sensitivity of wearable devices, paving the way for smarter and more responsive wearable technologies aimed at precision health management for older adults.

#### Enhanced Interaction with Targets

Because of their large surface area, nanomaterials provide numerous active sites to interact with target analytes, which increases the local concentration of analytes and leads to the high sensitivity of nanosensors.^16,115^ Therefore, designing nanosensors that enhance interaction with specific analytes offers an efficient strategy for improving the sensitivity of wearable devices for precision health care management. Particularly, porous nanomaterials, such as metal–organic frameworks (MOFs),^116^ covalent organic frameworks (COFs),^117^ and mesoporous carbon nanomaterials,^118^ have attracted much attention for their pore network structures and high specific surface areas, making them ideal candidates for high-performance wearable devices. Liu et al. constructed a COF-based wearable humidity sensor for monitoring the sleep respiratory behavior of sleep apnea patients (Figure 4D).^117^ The COF has ordered pore structures, which offer plenty of active sites to adsorb water molecules with rapid exchange capabilities, leading to high sensitivity and a rapid response to humidity. To demonstrate its practical feasibility, the researchers integrated the COF-based sensor with a circuit board, a mask device, and a smartphone for the real-time recording of normal breath, rapid breath, and breath apnea, showing promise for evaluating sleep function in older individuals. Qi et al. fabricated a wearable visual sensor based on fluorescent europium MOF (Eu-MOF) for the highly sensitive monitoring of cortisol.^116^ The large specific surface area of Eu-MOF provides numerous active sites that strongly interact with cortisol, resulting in the fluorescence quenching of Eu-MOF. This sensor exhibited an LOD as low as 10^–9^ M, showing great potential for the management of sleep disorders, depression, and chronic fatigue. In addition, because of their robust interaction with gas molecules, porous nanomaterials have also been utilized to construct sensitive wearable gas sensors.^119,120^ For example, Ren and collaborators reported a wearable NO_2_ textile sensor based on reduced graphene oxide–mesoporous ZnO nanosheet composite fibers.^119^ Benefiting from the outstanding adsorption and conductive properties of these nanocomposite fibers, the sensor showed a linear dynamic range of 0.2 to 5.0 ppm with an LOD as low as 43.5 parts per billion (ppb). This work offers an easy and cost-efficient way to construct gas sensors for the precise monitoring of respiratory-related diseases in older adults.

High sensitivity endows nanosensor-powered wearable devices with exceptional accuracy in monitoring subtle physiological and biochemical changes, thereby providing more precise health data that are crucial for early disease warnings. Additionally, these highly sensitive devices can detect minute variations in health parameters, resulting in richer and more comprehensive datasets that support in-depth health trend analysis for precision health management, benefiting older adult populations.

### Enhancing Device Specificity

#### Specific Interactions between Nanomaterials and Analytes

Nanomaterials exhibit properties, including flexible composition, adjustable size, and tunable surface chemistry, that are helpful for the rational design of nanosensors, facilitating specific interactions with analytes to enhance detection specificity.^17^ For example, MOFs, consisting of metal centers and organic ligands, possess highly ordered pore structures and active sites to bind with analytes.^121^ The metal centers and organic ligands can be flexibly tuned to mimic the active sites of natural enzymes, thus improving the specificity of analyte binding.^122^ Moreover, the surface of nanomaterials can be easily modified with functional groups, thereby enhancing the interaction with specific analytes to enhance detection specificity. The integration of these nanomaterial-based sensors with wearable devices offers great opportunities for enhancing detection specificity and guaranteeing data veracity for precision health management. Inspired by natural oxidase, Xia et al. developed the tryptophan- and histidine-modified MOF (HT-STAM-17-OEt) and reported its application for the specific detection of ascorbate in sweat (Figure 5A).^123^ Compared with unmodified MOF (STAM-17-OEt), HT-STAM-17-OEt specifically captured and adsorbed ascorbate from the complex components of sweat and promoted the electrooxidation of ascorbate. As a result, this MOF eliminated the interference of glucose, lactate, uric acid, ethanol, and cortisol in sweat, opening up new avenues for improving the specificity of wearable sensors for health care applications. In another work reported by Xie et al., a chiral molecule was utilized for the construction of a chiral MOF-based wearable sensor for detecting an active enantiomer.^124^ The chiral MOF was assembled with fluorescent and colorimetric substrates for dual-visual sensing. In the presence of L-lactic acid, the chiral MOF enantioselectively captured L-lactic acid, which caused the release of fluorescent and colorimetric substrates to generate colorimetric signals. Furthermore, the chiral MOF-based sensor was incorporated into a flexible membrane for the point-of-care monitoring of lactate during exercise, providing an efficient routine for the design of wearable devices for the monitoring of chiral markers.

**Figure 5:**
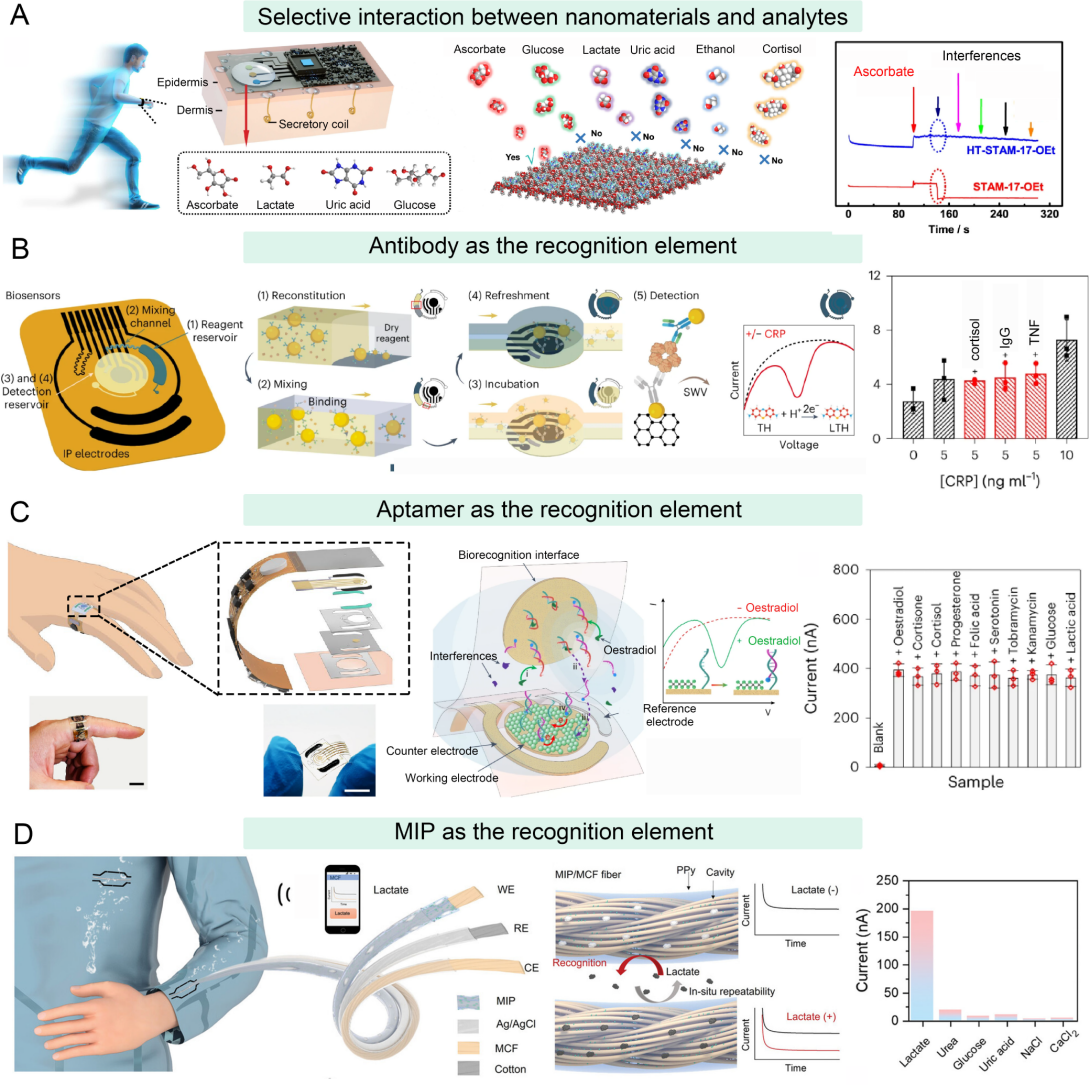
Integration of nanosensors to improve the selectivity of wearable devices. (A) Wearable sensor based on tryptophan- and histidine-modified MOF for the specific detection of ascorbate in sweat. Reproduced with permission under Creative Commons Attribution 4.0 International License from ref. 123. Copyright 2023, The Authors. (B) An antibody-functionalized electrochemical wearable device for the real-time monitoring of CRP in sweat. Reproduced with permission from ref. 126. Copyright 2023 Springer Nature. (C) A wearable aptamer nanosensor with self-regeneration properties for the long-term monitoring of a female hormone. Reproduced with permission from ref. 135. Copyright 2024 Springer Nature. (D) A MIP-based wearable fabric for the long-term monitoring of sweat lactate. MCF refers to multichannel fiber and PPy refers to polypyrrole. Reproduced with permission from ref. 139. Copyright 2024 Wiley-VCH.

#### Specific Interactions between Recognition Molecules and Analytes

In addition to interacting directly with analytes, nanomaterials with large surface areas can interact indirectly with analytes via functionalization with specific recognition molecules. The most common recognition molecules used in high-performance wearable devices are antibodies, functional nucleic acids, and MIPs.

With outstanding sensitivity and specificity, antibodies continue to play an irreplaceable role in immunoassays.^125^ Gao’s group developed an antibody-functionalized electrochemical wearable device for the real-time monitoring of C-reactive protein (CRP) in sweat (Figure 5B).^126^ For this device, gold nanoparticle-conjugated anti-CRP capture antibodies were added to a graphene electrode to generate a sensor array. In the presence of CRP, thionine/gold nanoparticle-conjugated detector antibodies are assembled onto the surface of the electrode due to the formation of an immunosandwich structure, leading to the generation of electrical signals from thionine. Moreover, the immunosensor was integrated with other modules, including sweat extraction, sweat sampling, reagent routing, signal processing, and wireless communication. Such integration enables wearable devices for the real-time measurement of CRP in sweat, revealing great promise for managing inflammatory diseases in older adult populations. Aran et al. reported an antibody-modified graphene transistor for the detection of aging-specific circulating exosomes via surface markers, including CD63 and CD151.^127^ This sensor showed satisfying results for the quantitative detection of purified exosomes from plasma, with an LOD as low as 2 × 10^4^ particles/mL, holding great promise for the diagnosis of aging-related diseases. Further studies have demonstrated that the orientation of antibodies plays an important role in the performance of wearable devices. Zhang et al. designed an antibody-oriented MOF immunosensor for a wearable device detecting cortisol in sweat.^128^ The antibodies were oriented with the fragment crystallizable region inside the MOF and the binding region exposed on the surface of the MOF. This orientation greatly improved antibody activity, resulting in a good linear detection range of 1 pg/mL to 1 μg/mL. Moreover, the wearable sensor exhibited high persistence in cortisol detection after 9 days, showing only a 4.1% decay in the detection signal. This work provides an efficient strategy for improving the stability of antibody-functionalized wearable sensors, exhibiting robust capability for long-term health monitoring, benefiting older adults.

Functional nucleic acids, including molecular beacons, aptamers, DNAzymes, and G-quadruplexes, can bind with specific analytes and cause conformational changes, leading to the generation of measurable signals.^129–132^ Among these, aptamers, selected by the systematic evolution of ligands by exponential enrichment (SELEX) technique, have received widespread attention for nanosensors because of their wide range of targets, including metal ions, small molecules, proteins, cells, and even tissues.^133^ Emaminejad et al. reported a wearable aptamer FET sensor array for monitoring cortisol in saliva.^134^ When bound with cortisol, the aptamer folds into a specific conformation and causes an electrical signal change in the FET. This sensor was combined with a liquid crystal display, a flexible substrate, and electronics to construct a watch that could directly read out cortisol concentrations. This wearable device can be used to monitor stress in older adults for the management of depressive disorders, Cushing’s disease, and Addison’s disease. Since the conformation of the aptamer can be tuned by pH, ionic strength, and environmental conditions, precisely regulating aptamer-targeted binding and dissociation offers an efficient strategy for the design of renewable sensors. For example, Gao’s group designed a wearable aptamer nanosensor with self-regeneration properties for the long-term monitoring of a female hormone (Figure 5C).^135^ In the presence of the target hormone, estradiol, aptamer molecules interact with estradiol and induce a strand displacement process to generate an electrical signal response. Then, after changing the buffer solution to deionized water, the aptamer–estradiol complex dissociates, leading to the recombination of aptamer and signal strands for the regeneration of aptamer nanosensors. This aptamer nanosensor was also integrated with microfluidic systems to enable sweat entry, collection, analysis, and outflow for automatic, *in situ* monitoring of estradiol. Furthermore, the sensor was incorporated into wearable electronic systems featuring iontophoresis, electric input and output, serial peripheral interface, and system-on-a-chip for application in human participants. This work offers a valuable example of designing renewable wearable sensors for the long-term monitoring of biomarkers in aging-related diseases.

MIPs are synthetic biomimetic receptors created by incorporating templates during polymerization to produce binding sites with high specificity and high affinity.^136^ With the advantages of low cost and mass producibility, MIPs have been widely used as the recognition element in biosensing.^137^ Zhang et al. reported a wearable MIP electrochemical patch for the measurement of sweat cortisol.^138^ In this electrochemical sensor, Prussian blue was used as a redox probe to generate measurable electric signals. When target cortisol binds with the MIP, electron transfer from Prussian blue to the electrode is impeded, decreasing the electric signal and thereby reflecting the cortisol concentration in sweat. These sensing results were displayed on a smartphone through wireless transmission, showing great promise for at-home monitoring of the mental status of older adults for the diagnosis of psychological diseases. In another work, He et al. reported an MIP-based wearable fabric for the long-term monitoring of sweat lactate, which may find extensive application in monitoring the daily health of older adults (Figure 5D).^139^

Nanosensor-powered wearable devices with high specificity provide a powerful platform for accurately eliminating background interference from the environment and matrices, enabling more reliable identification of abnormal changes in physiological and biochemical factors. This is significant for reducing both false-positive and false-negative rates, greatly enhancing the accuracy and effectiveness of health monitoring for older adults. This also leads to more scientific and precise decision-making in disease diagnosis and health management. The data generated by highly sensitive and specific wearable devices not only benefits users and health care providers but also offers a solid foundation for medical research and the development of new technologies, thus promoting overall improvement in managing the health of older populations.

## Machine Learning-Based Analytics of Aging-Related Health Data

Wearable devices and nanosensor technologies generate vast amounts of health data. Older adults experience a decline in physical function, leading to health data with more complex and frequent changes than health data associated with younger adults.^140^ Furthermore, the symptoms of health issues in older adults may be atypical, requiring the integration of health data from various dimensions to more accurately assess health status and diagnose conditions in older individuals.^141^ Therefore, it is of great importance to precisely and efficiently interpret the complex health data of older adults for health management in this population, but several barriers remain. For instance, the signals collected by wearable sensors typically require specific analysis and processing before they can be transformed into relevant health data.^142,143^ In addition, significant noise and interference can compromise the accuracy and reliability of these data.^144,145^ Moreover, recording individual data points often fails to fully capture the overall health status of older adults.^146^ In recent years, machine learning algorithms, including support vector machines, *k*-nearest neighbors, logistic regression, and artificial neural networks, have created new opportunities for addressing these data analysis challenges, playing a crucial role in precision health management for older adults.^146–148^ From large data sets, machine learning algorithms can extract features associated with specific health conditions, which can then be applied to disease diagnosis and health monitoring. Furthermore, machine learning is beneficial for parameter calibration in sensors, enhancing the reproducibility and accuracy of the acquired data. It can also integrate data obtained from different sensors to perform information mining, yielding deeper health insights for applications such as disease prediction and biomarker discovery. In this section, we discuss the role of machine learning algorithms in analyzing big health data for different aspects of wearable devices.

### Machine Learning Algorithms for Sensor Calibration

Health data collected from wearable sensors can be significantly influenced by the sensor’s condition, individual user differences, and environmental factors.^23^ Thus, for improved data accuracy and reliability, sensors must be calibrated using additional information. For this purpose, machine learning algorithms can analyze a vast array of environmental data, such as temperature, humidity, and pH, along with sensor parameters and user information. These algorithms can eliminate interference factors from the sensors, optimize the parameters within the models, and ultimately enhance the accuracy and robustness of the wearable system. For example, to ensure the robustness of wearable tactile devices against variations in individual sensors, Luo et al. developed a self-supervised machine learning algorithm for sensing calibration to improve device performance (Figure 6A).^149^ In detail, they first collected numerous tactile data points from sock and glove sensors to correlate the tactile responses at all of the sensing points across the entire body for calibration of these sensors. Then, a self-supervised network was adopted to calibrate the variation among individual elements, leading to improved reliability of the wearable devices. In another work, to eliminate interference caused by changes in the placement of the device, Han et al. developed a multilayer perceptron regression algorithm to analyze vibration waveform data at different positions for accurate calibration of the wearable sensors.^150^ Such a machine learning algorithm shows great promise for *in vivo* applications since body motion may have significant effects on the measurement of physiological and biochemical parameters by wearable devices. Additionally, Tian and collaborators employed a multivariate regression machine learning algorithm to precisely monitor core body temperature by a wearable thermal device.^151^ Data collected from the device was used to train the model, contributing to a prediction error of less than 0.25 °C. These calibrated data can more accurately represent health conditions, improving the effectiveness of health care management and enhancing the precision of disease diagnosis for older adult populations.

**Figure 6:**
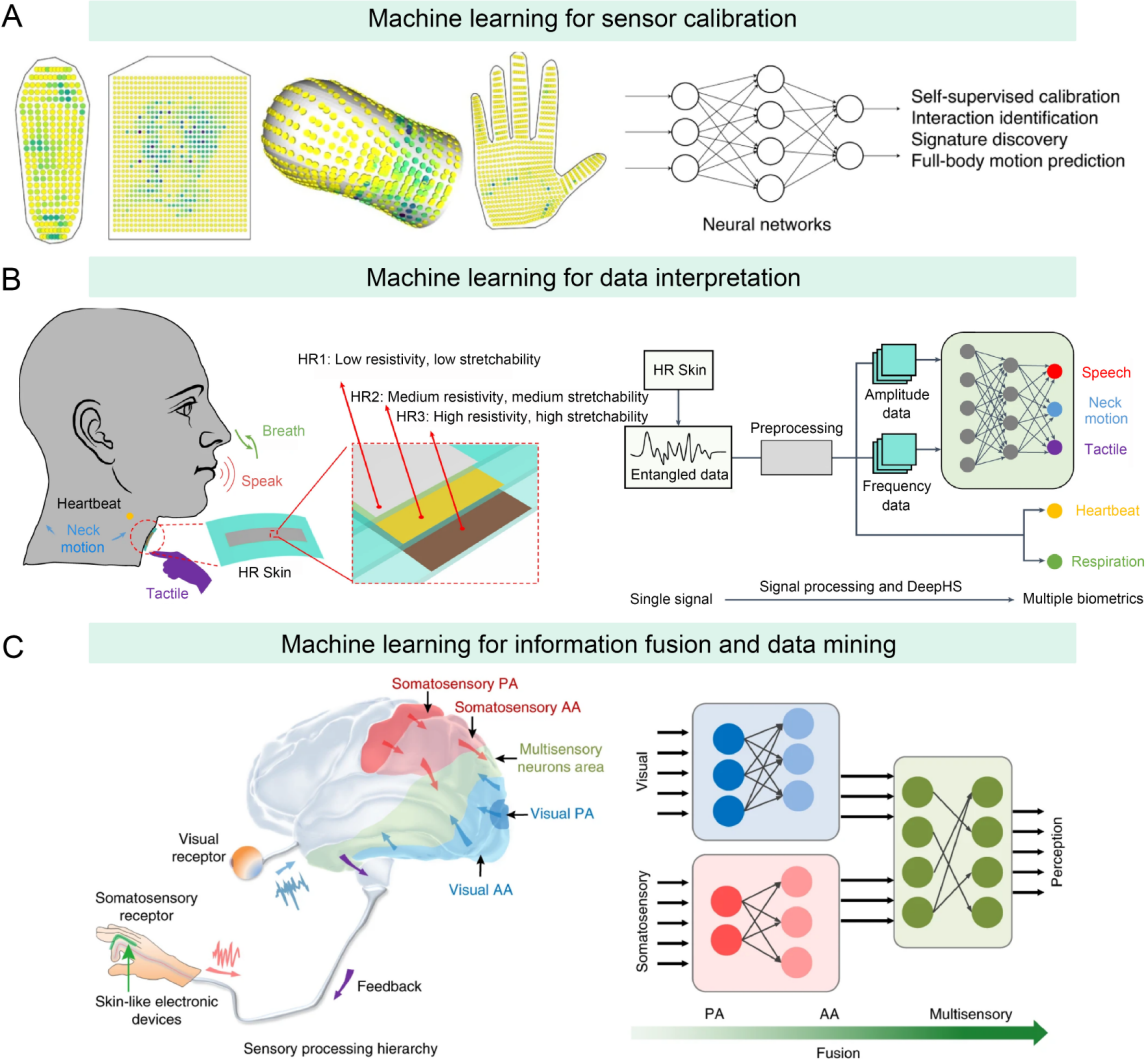
Machine learning for the analysis of data collected by wearable devices. (A) Self-supervised algorithm for sensing calibration. Reproduced with permission from ref. 149. Copyright 2021 Springer Nature. (B) A machine learning neural network based on frequency and amplitude signals to disentangle multiple biometrics from a signal sensor. Reproduced with permission from ref. 154. Copyright 2023 Springer Nature. (C) A bioinspired data fusion architecture that can integrate visual data and somatosensory data for accurate recognition of gestures. (PA, primary area; AA, associated area) Reproduced with permission from ref. 155. Copyright 2020 Springer Nature.

### Machine Learning Algorithms for Data Interpretation

Wearable devices generate vast amounts of health-related data and signals through continuous monitoring. Ordinary users often struggle to interpret the collected data, while professional analysis is hindered by high costs, time constraints, and an overwhelming workload.^152^ Moreover, these collected signals can be affected by distortion, noise, and baseline drift, which significantly impact the accuracy and reliability of analysis results.^23^ Therefore, integrating wearable devices with methods for real-time and automated data interpretation is essential for personalized health management. Specifically, raw signals obtained from wearable devices (especially those integrated with nanosensors) represent complex data streams that require sophisticated processing for effective machine learning analysis. These signals are inherently prone to noise from sources such as electrical interference, motion artifacts, and biological variability.^142,145^ Consequently, robust noise reduction techniques, including filtering (e.g., Kalman filters, wavelet transforms) or signal averaging, are essential to improve the signal-to-noise ratio and ensure data reliability—particularly crucial for detecting subtle health changes in older adults. Also essential are techniques for feature extraction, which transform the cleaned data into informative metrics or patterns that capture key characteristics relevant to the monitored health parameters.^146^ These techniques range from basic statistical measures (e.g., mean, variance) to advanced time-series analysis (e.g., Fourier transforms, principal component analysis).^147^ The high-quality extracted features then serve as the foundation for advanced machine learning algorithms discussed herein, enabling accurate pattern identification, health state classification, and predictive modeling. This comprehensive data processing pipeline is fundamental to realizing the full potential of wearable nanosensors for precision health management.

Machine learning excels at handling and analyzing complex data sets, enabling the automatic identification and analysis of relevant patterns. Furthermore, machine learning can recognize and eliminate noise and interference in the data, thereby improving its quality. By learning from extensive data sets, machine learning optimizes predictions and classifications, enhancing the accuracy and reliability of data interpretation for applications such as health monitoring and disease diagnosis. Exemplifying this, Zhang et al. designed a colorimetric wearable system to monitor tear biomarkers and integrated the system with a machine learning neural network to interpret the colorimetric data and a multichannel convolutional recurrent network to calibrate pH and temperature variations.^153^ By using the system, information on biomarkers, including vitamin C, pH, Ca^2+^, and proteins, was accurately obtained, displaying huge potential for health monitoring and disease diagnosis. In another work, Cheng and collaborators developed a machine learning neural network based on frequency and amplitude signals to disentangle multiple biometrics from a single sensor (Figure 6B).^154^ They constructed a single integrated sensor composed of 3 resistive skin layers based on different active nanomaterials to generate 1 resistive signal. The machine learning algorithm was trained to identify up to 11 activities with high accuracy—more than 90%—providing an efficient strategy for remote health care. Compared with data analysis achieved by wirelessly transporting raw data into additional computational equipment, analyzing the data locally has the advantages of enhanced latency and security. In this regard, Moin et al. developed an in-sensor adaptive machine learning algorithm to analyze the collected data from a wearable biosensing system for the recognition of hand gestures.^53^ This hyperdimensional algorithm was used to train the model and calibrate variations, including different arm positions and sensor replacement. Consequently, the system recognized 13 kinds of hand gestures with an accuracy of 97.12% without the requirement of additional computational devices. In general, algorithms like these can be utilized to recognize health conditions such as a decline in physical activity, a metabolic imbalance, or a sleep disorder, which could be helpful for promptly identifying potential health risks in older adults.

### Machine Learning Algorithms for Information Fusion and Data Mining

Wearable devices can integrate a series of sensors to collect information from different body parts, utilizing diverse signal modes and capturing various biomarkers and physiological characteristics.^146^ A comprehensive analysis of these data provides a more complete picture of the health status of older individuals and populations, facilitating precision health management for older adults. Machine learning offers effective methods for analyzing the diverse types of data collected from various wearable sensors, such as smartwatches, rings, and heart rate monitors. By fusing data from different sensors, machine learning algorithms can identify and eliminate redundant information, enhancing the efficiency of data analysis and improving analysis accuracy. For example, Chen’s group developed a bioinspired data fusion architecture that can integrate visual data and somatosensory data for the accurate recognition of hand gestures (Figure 6C).^155^ The architecture was made up of 3 neural networks, namely, a sectional convolutional neural network mimicking visual processing, a multilayer neural network resembling somatosensory processing, and a sparse neural network for the high-level fusion of these interactions. Compared with solely visual-based recognition and solely somatosensory-based recognition, this integrated approach effectively eliminated noise, as well as over- and under-exposures, reaching an accuracy of 100% by using a custom data set, even against complex backgrounds. This data fusion architecture presents an illustrative example of the integration of data from multiple sensors to improve the accuracy of wearable devices for precision health management.

Machine learning also excels at uncovering associations among different types of data. By leveraging these insights, personalized health recommendations can be provided to users, enabling predictions of future health risks. As an example, glucose, ECG, and accelerometer data collected by multiple sensors were integrated by a machine learning algorithm to predict diabetes.^156^ Compared with data from a single sensor, combining data from the 3 sensors significantly increased the rate of diabetes prediction, with an accuracy of 98.2%, showing great promise for the management of diabetes. Additionally, through population data mining on the chemical signature of disease status, machine learning holds significant potential for biomarker discovery and precise personalized health guidance, which is not only critical for the accurate diagnosis and timely treatment of aging-related diseases but also highly valuable for formulating effective rehabilitation programs.^157^

In general, nanosensor-derived data enable clinicians to deliver personalized, proactive, and adaptive health management solutions for older adults. This approach enhances preventive care, improves safety, and elevates the quality of care, all while providing clinicians with more transparent and precise decision-making support. Moreover, machine learning enhances the design of precision health sensors for older adults by analyzing data to pinpoint which physiological and biochemical indicators—such as heart rate variability, sleep architecture, or mobility metrics—most effectively forecast health decline or emergencies. By identifying these critical signals, machine learning directs engineers to prioritize and optimize sensor modalities, sensitivity, and data integration for maximum clinical relevance. Furthermore, machine learning can illuminate unique comorbidity patterns and daily activity variations within older adult populations, influencing the development of user-friendly and multifunctional sensors. Real-world data, continuously analyzed by machine learning algorithms, allow for iterative improvements in sensor accuracy and usability, ultimately ensuring effective personalized monitoring, timely interventions, and superior health outcomes for older adults (Table 3).

**Table 3:** Common Machine Learning Models and Corresponding Applications for Precision Health Management Benefiting Older Adults

Type	Models	Clinical utility	Algorithms	Limitations	Applications for aging-related issues
Supervised learning	Regression	Estimating a numeric and continuous clinical outcome	Linear regression, decision tree regression, random forest regression	Linearity assumption, oversimplification, ignoring individual differences	Blood pressure estimation,^158^ glucose monitoring,^159^ sleep quality assessment^160^
	Classification	Categorizing data into distinct classes or groups	Support vector machine, logistic regression, deep learning (i.e., convolutional neural network, physics-informed neural network)	Loss of granularity, overfitting, difficult to interpret	Arrhythmia detection,^161^ fall detection, cognitive impairment screening^162^
	Forecasting	Predicting future health states	Prophet, recurrent neural network (i.e., long short-term memory network)	Stationarity assumptions, model complexity, nonlinearity	Prediction of stroke and arrhythmias, blood pressure trend prediction, fall prediction^163,164^
Unsupervised learning	Clustering	Grouping individuals with similar characteristics, behaviors, or health patterns	K-means clustering, spectral clustering, hierarchical clustering	Defining similarity, sensitivity to initialization, computational cost	Outlier removal from wearable data^165,166^
	Dimension reduction	Simplifying complex, high-dimensional data sets	Principal component analysis, linear discriminant analysis, autoencoders	Information loss, difficult to interpret, nonlinearity	Feature selection, data compression, and noise reduction in specific applications^167,168^
Reinforcement learning	Automated decision-making	Learning optimal personalized interventions	Q-learning	Ethical concerns, lack of transparency, bias amplification	Adaptive exercise recommendations, medication adherence optimization, fall prevention training^169^

## Proactive Health Management, Based on Data Analytics, for Older Adults

The integration of wearable device-collected health data with machine learning algorithms offers a powerful approach to proactively prevent adverse health events, creating a closed-loop system of “diagnosis-analysis-prevention” that benefits older adults. This is increasingly realized through a structured framework for proactive health management. First, individual-level physiological, biochemical, behavioral, and environmental data are comprehensively acquired via unobtrusive wearables, Internet of Things devices, electronic health records, and patient-reported outcomes.^170^ These heterogeneous data streams are then consolidated into a secure, interoperable, and accessible data platform. Subsequently, advanced machine learning algorithms, including deep learning models, analyze the integrated dataset to identify latent patterns predictive of aging-related health risks.^10^ Risk stratification algorithms categorize individuals based on their probabilistic risk profiles for adverse events. Informed by these individualized risk scores, personalized intervention strategies are designed and deployed, encompassing artificial intelligence-driven coaching systems, tailored exercise regimens, medication adherence support, and remote physiological monitoring tools.^171,172^ A continuous feedback loop, incorporating real-world outcome data, iteratively refines both the predictive models and intervention protocols. The final phase integrates all components into a scalable, user-friendly platform accessible to older adults, caregivers, and health care providers, emphasizing adherence to regulatory standards, data privacy protocols, and ethical guidelines to ensure responsible and widespread implementation. Ultimately, this data-driven, precision-oriented approach aims to mitigate aging-related health risks, optimize functional status, and promote enhanced well-being in the older adult population.^173^

Wearable sensor-powered machine learning models can forecast the probability of specific health events in older adults, including falls, cardiovascular incidents, respiratory exacerbations, and cognitive decline. Upon detection of elevated risk via real-time wearable device data, the system issues alerts, prompting timely intervention measures by the user, their family members, or their healthcare providers.^174^ For instance, in the event of a fall or other emergency, an automated call can be initiated to emergency services, facilitating a rapid response.^174^

In addition, wearable devices leveraging collected data generate personalized health profiles for older adults, accounting for individual health conditions and lifestyle habits.^175^ Subsequently, machine learning algorithms tailor specific intervention strategies, encompassing exercise, diet, medication, and sleep.^176^ These strategies may include recommending suitable exercise types, intensities, and frequencies to enhance physical fitness and prevent falls; providing personalized dietary guidance to manage weight and reduce the risk of chronic diseases; issuing reminders for scheduled medication intake and monitoring drug efficacy and potential side effects; and offering evidence-based suggestions for improving sleep quality, such as adjusting sleep schedules and optimizing the sleep environment.

Integrating wearable devices, machine learning algorithms, and personalized interventions into a comprehensive health management platform empowers older adults, their family members, and their health care providers with easy access to health data, alerts, and tailored recommendations.^177^ A key benefit is facilitating remote consultations with physicians for professional guidance.^178^ By leveraging wearable devices and machine learning, continuous monitoring and personalized risk prediction can be achieved, enabling early warnings and proactive intervention suggestions prior to the manifestation of adverse health events. This proactive health management model facilitates early detection of potential risks, reduces morbidity and mortality rates among older adults, improves their quality of life, and alleviates the burden on health care systems, thereby offering significant personal and societal benefits, including economic ones.

The integration of wearable technology and machine learning into a comprehensive health management platform offers a powerful, proactive approach to older adult care by enabling personalized risk prediction, early intervention, and improved overall well-being. This data-driven strategy not only enhances the quality of life for older adults but also alleviates the burden on healthcare systems through early detection and preventive measures.

## Challenges and Perspectives

Applying big data to health care is driving the industry to shift from a passive treatment model to an active, integrated “diagnosis-analysis-prevention” model, highlighting the importance of precision health management for older adults. As vital tools for managing the health of older adults, with a wide range of application scenarios, wearable devices can collect large volumes of health data with robust real-time and continuous capabilities. Nanosensors can effectively enhance the sensitivity and specificity of health indicators monitored by wearable devices, thereby improving the quality of the collected health data. Building on this foundation, machine learning provides a powerful tool for analyzing large health datasets. The combination of wearable devices, nanosensors, and machine learning offers an efficient and accurate solution for precision health management, benefiting the older adult population. In this perspective, we discuss the role of wearable devices, nanosensors, and machine learning in massive data collection, data quality enhancement, and data analysis, shedding light on the application of big data for precisely managing the health of older adults. Despite significant research progress, this field still holds vast potential for further development.

Currently, the parameters that can be monitored by wearable devices for data collection remain limited. For example, biophysical information is primarily confined to signals from the skin surface. Developing deeper tissue-sensing technologies, such as ultrasound and magnetic resonance imaging, will help obtain more health information and provide insight into the health conditions of older adults.^179,180^ Similarly, the biochemical information currently collected is mainly limited to electrolytes and metabolites, although several wearable devices in development have started to monitor protein and nucleic acid concentrations. As antibody- and nucleic acid-sensing technologies continue to be incorporated into wearable devices, an increasingly comprehensive range of biochemical data will be collected, accelerating the discovery of biomarkers. The extension of monitoring capabilities to include signals from deeper tissues and a more comprehensive analysis of biochemical markers also represents a significant advancement in the early detection and characterization of aging-related diseases. This expanded approach offers the potential to elucidate the underlying mechanisms and progression of conditions such as cardiovascular disease, neurodegenerative disorders, and cancers, ultimately leading to earlier intervention and improved patient outcomes. Currently, single wearable devices are limited not only by the type of information they collect but also by the number of indicators they can monitor, making it challenging to provide comprehensive health information for older adults. This limitation could be addressed by wearable clothing, a promising option for collecting diverse signals to obtain a more holistic understanding of the body’s condition. Moreover, as application scenarios and user needs become more defined, the development of wearable devices can also prioritize specialized designs for specific diseases and daily health management.

Current nanosensors also have limitations, with parameters such as regenerable performance, temporal stability, and multiplexing being the most important. This is particularly true for antibody- and nucleic acid-based sensors, in which the specific binding of antibodies and nucleic acids to their targets presents significant challenges in achieving regeneration while ensuring reproducibility and stability in the recognition process. This aspect is crucial for the long-term monitoring of the performance of wearable devices. At the same time, the ability to simultaneously detect multiple disease biomarkers is significant for enhancing health monitoring and improving the accuracy of disease diagnosis, taking into account the complexity of aging-related diseases. However, the corresponding wearable sensors are still under development. Because of their broad target range, nucleic acid probes, integrated with microfluidic biochips, hold considerable potential for the development of wearable sensors aimed at the simultaneous determination of different types of biomarkers.^181^ Improved performance in these parameters directly translates into more reliable and clinically relevant data, facilitating long-term monitoring of chronic conditions and the simultaneous detection of multiple biomarkers. This capability is essential for realizing accurate diagnoses and personalized treatment strategies specifically tailored to the unique needs of older adults. Beyond core detection performance, ensuring long-term stability and biocompatibility for materials in continuous contact with skin and body fluids is paramount, requiring robust materials and surface modifications to prevent degradation and adverse reactions. Minimizing power consumption is crucial for extended battery life, requiring the development of energy-efficient sensor designs. Furthermore, environmental factors, particularly motion artifacts, significantly impact signal accuracy, necessitating sophisticated signal processing algorithms and robust sensor engineering to maintain data fidelity. Overcoming these interconnected hurdles is essential for providing reliable data to machine learning platforms, ultimately enabling effective precision health management for older adults. These challenges highlight the need for continued research and multidisciplinary collaboration in materials science, sensor design, and data analytics to realize the full potential of these technologies.

Although machine learning is a powerful tool for data analysis, it also presents unique challenges. Wearable sensors can collect massive amounts of data, which are essential for continuously optimizing and improving the accuracy of machine learning algorithms, enabling these algorithms to provide more precise health management results and play a broader role in disease diagnosis and biomarker discovery. However, processing this massive amount of health data creates privacy and security issues, which cannot be overlooked. The design of safe encryption and data protection mechanisms through machine learning algorithms will offer an efficient way to ensure the security of user data. Interpretability is also important for the successful integration of machine learning models into health care. As algorithms leverage nanosensor data to drive health predictions, transparency regarding their reasoning is crucial for building trust and supporting informed decisions by clinicians, caregivers, and patients. Employing interpretable techniques, such as feature importance analysis and tools, unveils the key sensor-derived features that influence predictions and enable validation, error analysis, and personalized care adjustments. Therefore, prioritizing model interpretability is vital not only for regulatory compliance and clinical buy-in but also for empowering older adults and their care teams to confidently embrace and benefit from intelligent wearable health technologies. Advanced algorithms can then effectively analyze complex, high-dimensional data streams to provide prognostic assessments and personalized therapeutic guidance. This capability is particularly valuable for clinicians managing the multifaceted health needs of older adult patients, enabling both predictive modeling of adverse health events, such as falls or disease exacerbations, and proactive interventions.

The “diagnosis-analysis-prevention” loop for precision health management also presents significant challenges. Paramount among these are data privacy and security, necessitating stringent safeguards to protect sensitive personal information derived from wearable sensors and connected devices. Furthermore, scalability and seamless integration with existing healthcare infrastructures require addressing interoperability issues and navigating intricate regulatory frameworks. Nevertheless, successful implementation of this closed-loop system offers considerable promise, enabling proactive, personalized care that facilitates early disease detection and prevention, reduces hospital readmissions, and improves the overall quality of life. Remote monitoring and management of chronic conditions can empower older adults to maintain independence and facilitate aging in place. Critical ethical considerations, including informed consent procedures and data ownership rights, must guide future development, ensuring that deployed technologies promote human well-being and equitable access to care.

Commercializing wearable solutions for older adult health management faces numerous hurdles. Economic barriers, including high costs and limited insurance coverage, create financial strain. User-related challenges involve technical difficulties, comfort issues, and resistance stemming from privacy concerns and data anxiety. Furthermore, technical and systemic obstacles persist, such as ensuring data quality despite variability, tackling data security and interoperability, navigating regulations and ethics, delivering truly personalized services, and addressing adherence and battery life limitations. Overcoming these multifaceted barriers is essential for widespread adoption and commercial success. A focus on affordability, user-friendliness, data security, and reliable performance is crucial for bridging the gap between promising technologies and practical, accessible solutions for older adults. In the foreseeable future, the rapid development of wearable devices, nanosensing technologies, and machine learning will drive significant transformations in the field of big health data, providing effective solutions for precision health management that benefit older adults.

## Vocabulary

Wearable devices: Smart electronic gadgets designed to be worn on the body or integrated into clothing, continuously and noninvasively collecting a variety of real-time user data, including physiological metrics, activity levels, and environmental factors.
Nanosensors: Sensors that leverage the unique physical and chemical properties of nanoscale materials (e.g., nanoparticles, nanowires) achieve highly sensitive and specific detection of minute substances or weak physical signals.
Big health data: Massive, diverse, rapidly generated, and potentially high-value but low-density collection of information originating from healthcare, health management, and life sciences.
Machine learning: A subset of artificial intelligence that enables computer systems to automatically identify patterns, make predictions, and drive decisions by learning from data, rather than through explicit programming.
Health management: A systematic process aimed at promoting well-being, preventing illness, and improving quality of life through comprehensive health risk assessment, continuous monitoring, and scientific intervention for individuals or populations.
